# Effects of dietary *Antrodia cinnamomea* fermented product supplementation on metabolism pathways of antioxidant, inflammatory, and lipid metabolism pathways-a potential crosstalk

**DOI:** 10.5713/ajas.19.0393

**Published:** 2019-08-26

**Authors:** M. T. Lee, W. C. Lin, L. J. Lin, S. Y. Wang, S. C. Chang, T. T. Lee

**Affiliations:** 1 Department of Animal Science, National Chung Hsing University, Taichung 402, Taiwan; 2 School of Chinese Medicine, College of Chinese Medicine, China Medical University, Taichung 402, Taiwan; 3 Department of Forestry, National Chung Hsing University, Taichung 402, Taiwan; 4 Kaohsiung Animal Propagation Station, Livestock Research Institute, Council of Agriculture, Kaohsiung 912, Taiwan; 5 The iEGG and Animal Biotechnology Center, National Chung Hsing University, Taichung 402, Taiwan

**Keywords:** *Antrodia cinnamomea*, Boiler Chickens, Nuclear Factor (Erythroid-derived 2)-like 2 (Nrf2), Nuclear Factor-kappa B (NF-κB), Lipid Metabolism

## Abstract

**Objective:**

This study was conducted to fathom the underlying mechanisms of nutrition intervention and redox sensitive transcription factors regulated by *Antrodia cinnamomea* fermented product (FAC) dietary supplementation in broiler chickens.

**Methods:**

Four hundreds d-old broilers (41±0.5 g/bird) assigned to 5 groups were examined after consuming control diet, or control diet replaced with 5% wheat bran (WB), 10% WB, 5% FAC, and 10% FAC. Liver mRNA expression of antioxidant, inflammatory and lipid metabolism pathways were analyzed. Prostaglandin E2 (PGE_2_) concentration in each group were tested in the chicken peripheral blood mononuclear cells (cPBMCs) of 35-d old broilers to represent the stress level of the chickens. Furthermore, these cells were stimulated with 2,2′-Azobis(2-amidinopropane) dihydrochloride (AAPH) and lipopolysaccharide (LPS) to evaluate the cell stress tolerance by measuring cell viability and oxidative species.

**Results:**

Heme oxygenase-1, glutathione S-transferase, glutamate-cysteine ligase, catalytic subunit, and superoxide dismutase, and nuclear factor (erythroid-derived 2)-like 2 (Nrf2) that regulates the above antioxidant genes were all up-regulated significantly in FAC groups. Reactive oxygen species modulator protein 1 and NADPH oxygenase 1 were both rather down-regulated in 10% FAC group as comparison with two WB groups. Despite expressing higher level than control group, birds receiving diet containing FAC had significantly lower expression level in nuclear factor-kappa B (*NF-κB*) and other genes (inducible nitric oxide synthase, tumor necrosis factor-α, interleukin-1β, nucleotide-binding domain, leucine-rich-containing family, pyrin domain-containing-3, and cyclooxygenase 2) involving in inflammatory pathways. Additionally, except for 3-hydroxy-3-methyl-glutaryl-coenzyme A reductase that showed relatively higher in both groups, the WB, lipoprotein lipase, Acetyl-CoA carboxylase, fatty acid synthase, fatty acid binding protein, fatty acid desaturase 2 and peroxisome proliferator-activated receptor alpha genes were expressed at higher levels in 10% FAC group. In support of above results, promoted Nrf2 and inhibited NF-κB nuclear translocation in chicken liver were found in FAC containing groups. H_2_O_2_ and NO levels induced by LPS and AAPH in cPBMCs were compromised in FAC containing diet. In 35-d-old birds, PGE_2_ production in cPBMCs was also suppressed by the FAC diet.

**Conclusion:**

FAC may promote Nrf2 antioxidant pathway and positively regulate lipid metabolism, both are potential inhibitor of NF-κB inflammatory pathway.

## INTRODUCTION

Oxidative reactions serve as the fundamental part of numerous biochemical pathways and cell functions. For instance, reactive oxygen species (ROS) are not only by-products of normal oxygen metabolism in aerobic organisms, but acting as signaling molecules regulating physiological and biological process, therefore, redox-sensitive gene expression regulated by ROS has become increasingly appreciated [1]. Once the levels of reactive species exceed the neutralizing ability of antioxidant system, such imbalance thereafter causes oxidative stress, leading to the activation of stress-sensitive intracellular signaling pathways, consequently cause damage to cellular macromolecules responsible for pathological conditions and diseases [2].

Genetic selection toward fast growth rates in order to have lean and large breast muscles make domestic avian, especially broilers, are extremely susceptible to oxidative stress [3]. Furthermore, free radical can be produced via endogenous metabolism and exogenous stimuli, suggest that these reactive species productions in the body is substantial, and a variety of biological molecules can be easily damaged once without proper protection [4,5]. Consequently, restricting oxidative processes is of prime importance for decent animal health, growth, production and economic feasibility.

Since nutrition is one of the most pertinent external factors in the prevention of diseases development, and the restricted ability of adaptive systems in animals to conquer oxidative stress, dietary supplementation with materials possessing antioxidant capacity become a decent candidate for external help in boosting endogenous defense mechanisms by influencing the redox sensitive molecules and thus identifying an important level of gene-nutrition interaction [6]. Several studies have demonstrated that as being secondary messengers to influence gene expression, ROS signaling route could be affected directly or indirectly by molecules attending lipid metabolism [7]. Moreover, oxidative stress induced by ROS accumulation is also considered to impair the optimal functioning of the immune system [8], showing the importance to target molecules involved in these signal transduction network.

Since many components of the cell signaling network would converge on transcription factors, by tracing those upstream and downstream molecules, we could simply clarify the pathways involved in specific conditions. Based on the urgency to tackle the severity of oxidative stress in animal production, focusing on oxidative-related genes are of importance. Therefore, aiming at redox-sensitive transduction pathways such as nuclear factor (erythroid-derived 2)-like 2 (Nrf2), nuclear factor-kappa B (NF-κB), and peroxisome proliferator-activated receptor alpha (PPARα) turns out to be a promising way.

Nrf2 is the principal transcription factor regarded as a mas ter regulator for the antioxidant response via translocating to bind to the antioxidant response element located in the promoter region of genes encoding numerous phase II detoxifying antioxidant enzymes and related stress-responsive proteins. These include glutathione S-transferase (GST), heme oxygenase-1 (HO-1), glutathione peroxidase (GSH-Px), and glutamate cysteine ligase (GCL) that play key roles in cellular defense by enhancing the removal of cytotoxic electrophiles or ROS [9]. In comparison with the mechanism of direct antioxidants, antioxidant actions driven by Nrf2 activation are regarded as indirect antioxidant regulation. Since indirect antioxidants act through gene expression, their physiological effects last longer than those being exerted by direct antioxidants. Inflammatory responses to a wide variety of stimuli mainly attribute to up-regulation of the proinflammatory transcription factor- NF-κB. Since it is a kind of redox-sensitive transcription factors, NF-κB responses to a number of stimuli including ROS [10]. After activation, NF-κB would translocate to nucleus, and then induces the expression of different inflammatory cytokines and chemokines, enzymes such as cyclooxygenase (COX2) and nitric oxide synthase (NOS), and many other genes related to cellular transformation, invasion, metastasis and inflammation [11]. Additionally, PPARs are transcription factors belonging to the nuclear receptor superfamily, among the three subtypes, PPARα activation is associated with a number of cellular process including lipid and lipoprotein metabolism, antioxidant defense, and anti-inflammatory response, being viewed as an attractive target regulated by nutrient [8]. Considering the absence of Nrf2 is associated with increased oxidative stress, leading to amplification of cytokine production, as NF-κB is more readily activated in oxidative environments [12], and that PPARα-mediated activation of lipid metabolism is capable of positively activating cellular protective Nrf2 pathways [13] and repressing inflammatory genes through negatively interfering transcriptional activity of NF-κB [14]; applying materials possessing the ability to affect these signaling pathways would be promising diet supplements in poultry husbandry for their long term effects and effectiveness.

In our separate manuscript, results demonstrated that di etary supplementation of fermented wheat bran by *Antrodia cinnamomea* (solid-state fermented wheat bran by *Antrodia cinnamomea* for 16 days, FAC) could improve the antioxidant capacity of chickens, better intestinal microflora and serum lipid profile. Moreover, due to the close related association among lipid metabolism, antioxidant and inflammatory response, the present study aims to study the effects of FAC on the components of signal transduction network in the above three parts, in order to elucidate the underlying regulatory control mechanisms.

## MATERIALS AND METHODS

### Animal management

Four hundred 1-d-old male broiler chickens (Ross 308) were evenly divided by weight (approximately 41±0.5 g/bird) and then randomly allocated to one of the five treatments. Each treatment group had four replicates per pen, with 20 birds per pen (totaling 80 birds per treatment). The temperature was maintained at 34°C±1°C until the birds reached 7 days of age; it was then gradually decreased to 26°C±1°C until the birds reached 21 day of age. After this point, the broilers were maintained at room temperature (RT, approximately 27°C). The experiment was conducted at the ranch of National Chung Hsing University, Taiwan, and the experimental protocol was approved by the Animal Care and Use Committee (IACUC NO:102-126). The birds in the control group received corn-soybean meal basal diet; the 5% WB group was fed the basal diet with 5% replacement of wheat bran (WB); the 10% WB group was fed the basal diet with 10% replacement of WB; the 5% FAC group was fed the basal diet with 5% replacement of solid-state fermented wheat bran by *Antrodia cinnamomea* (AC), AC mycelia was kindly provided from Department of Forestry at National Chung Hsing University, Taichung, Taiwan (Dr. Sheng-Yang Wang lab), for 16 days (FAC); and the 10% FAC group was fed the basal diet with 5% replacement of FAC (Table 1). All birds received starter (1 to 21 days of age) and finisher (22 to 35 days of age) diets *ad libitum* and had free access to water. The proximate composition of the diets was analyzed according to the AOAC [14]. Crude protein, crude fat, ash, and acid detergent fiber levels were determined using methods 990.03 (Kjeldahl N×6.25), 945.16, 967.05, and 973.187, respectively; the results showed no major deviations from the calculated values. During the entire experimental period (35 days), the diets were formulated to meet the nutrients requirements suggested by Ross Broiler Management Manual [15] and NRC [16].

### Sample collection

At 21 and 35 d, eight birds (two birds per replicate) were randomly selected from each treatment group for sampling. Blood samples were collected via wing-vein puncture into a tube containing 1% ethylenediaminetetraacetic acid (EDTA). The samples were then centrifuged at 3,000×g for 10 min to obtain the serum, and the aliquots were transferred into microfuge tubes. Sera were kept on ice and protected from light to prevent any oxidation during sample collection. Samples were stored at −20°C until analysis. The birds were euthanized by electrical stunning for extermination, and then the abdominal cavities were opened for liver collection.

### Liver RNA isolation and quantitative reverse transcription-polymerase chain reaction

The procedure for RNA isolation and purification were performed followed the manual of RNA isolation kit (AllBio Science, Inc., Taichung, Taiwan). In brief, after washed in ice-cold physiological saline, approximately 0.5 g of each tissue was incubated in the binding buffer provided in the kit, and ground with a pestle and mortar. Seventy-five μL protease supplied, and mix the solution thoroughly by vortexing. After incubate for 20 minutes at 56°C, equal volume of RNase-free 70% ethanol was added to the lysate. The solution was vortexed thoroughly to disperse the precipitate which may form after adding ethanol. Centrifuge briefly and add all the lysate into the spin columns, and then centrifuge again at 12,000×g for 30 seconds, then discard the flow through. Four hundred μL clean buffer was added to the spin column, do the centrifuge again at 12,000×g for 30 s, and then discard the flow through. Five hundred μL wash buffer was then added into the spin column, perform the centrifuge on the empty column at 12,000×g for 2 min at room temperature in order to remove ethanol residue and then air-dry the column matrix for 20 min. Place the spin column into a clean 1.5 mL RNase free tube. Add 50 μL of RNase-free water into the spin column matrix and incubate at room temperature for 1 min. After done the final centrifuge at 12,000×g for 2 min to elute RNA, the isolated RNA was stored at −80°C for the subsequent analysis.

RNA concentration was determined by spectrophotometry and diluted to 50 ng/μL. Total RNA concentration and purity, cDNA synthesis and quantitative polymerase chain reaction (qPCR) analysis (StepOnePlus Real-Time PCR System. Roche Diagnostics Ltd., Rotkreuz, Switzerland) were determined as per the methods of Lin et al [17]. Gene-specific primers were designed based on the genes of *Gallus gallus* (chickens); and Table 2 lists the features of the primer pairs. After the normalization of gene expression data, the means and standard deviation were calculated for samples from the same treatment groups.

### Protein extraction and Western blot analysis

For Western blot analysis, nuclear and cytosolic extracts of the livers were prepared by Nuclear Extraction Kit according to the manufacturer’s instructions (FIVEphoton Biochemicals, San Diego, CA, USA). After quantification of protein concentration, equal amounts of proteins were separated by 10% sodium dodecyl sulfate-polyacrylamide gel electrophoresis (SDS-PAGE) and transferred to poly-vinylidene difluoride (PVDF) membrane (GE Healthcare Life Science, Pittsburgh, PA, USA). Upon protein transfer, the blots were washed five times for 5 min in phosphate-buffered saline (PBS) and blocked with Blocking Buffer for 1 h prior to the application of the primary antibody. Chicken antibodies against NF-κB and Nrf2 were purchased from Abcam (Cambridge, UK). The primary antibody was diluted (1:1,000) in the same buffer containing 0.05% Tween-20. The PVDF membrane was incubated overnight at 4°C with the primary antibody. The membrane was then washed five times with 0.05% PBST (PBS and Tween 20) for 5 min before being incubated with the corresponding secondary antibodies for 1 h at room temperature. Specific binding was detected using hydrogen peroxide as substrates.

For the first animal trial, Protein loading was controlled using a monoclonal-mouse antibody against β-actin antibody (Biorbyt or b40714). For the second animal trial, cytosolic and nuclear protein loading was controlled using a monoclonal-rabbit antibody against glyceraldehyde 3-phosphate dehydrogenase antibody (GeneTex, #GTX100118) and p84 (GeneTex, #GTX102919), respectively.

Band intensities of the proteins were quantified by densi tometric analysis using an image analysis system (Image J; National Institute of Health, Bethesda, MD, USA). Samples were analyzed in triplicate; a representative blot is shown in the respective figures.

### Chicken peripheral blood mononuclear cells isolation

Whole blood of chicken was collected via wing-vein using a hypodermic syringe and inserted into tubes containing EDTA. The blood was gently layered on to Ficoll-Paque Plus and centrifuged at 200×g for 10 min. Chicken peripheral blood mononuclear cells (cPBMCs) were collected from the gradient interface; the plasma suspension was combined and washed three times with PBS and then centrifuged at 200×g for 10 min. After the suspension was removed, RPMI-1640 was used as the solvent for adjusting cell count to 10^8^ cell/mL, which were pipetted 2 mL cell suspension into 6 well plates and cultured at 37°C in 5% CO_2_ mixed with 95% air in an incubator for 2 h. After incubation, the cells were treated with 2,2′-Azobis(2-amidinopropane) dihydrochloride (AAPH, 10 mM) or lipopolysaccharide (LPS, 100 ng/mL) for 24 h. Pipetting the whole culture liquid into a sterilized tube, and centrifuged at 200×g for 10 min. One mL Trizol reagent was added and the mixture was stored at −80°C.

### PrestoBlue assay for evaluating cell viability

The PrestoBlue agent is a highly sensitive and resazurin based reagent for assessing cell viability. The following procedures was performed according to the manufacturer’s protocol. Isolated cPBMCs were seeded at a density of 1×10^7^ cells/well in 96-well plates for 2 h. After incubation, the cells were treated with AAPH (10 mM) or LPS (100 ng/mL) for 24 h. After the treatment, the cells were washed by PBS. One hundred μL PrestoBlue solution was added to each well and the plates were incubated at 37°C for 1 h. After the incubation, 100 μL of the PrestoBlue solution from each well was transferred to a new well in 96-well plate. The absorbance was recorded at 570 nm and 600 nm, and then counted the viability rate according to the manufacturer’s instruction, and the cell viability was expressed as a percentage relative to the cells untreated with plant compounds.

### Measurement of nitric oxide content in chicken peripheral blood mononuclear cells

Nitric oxide (NO) production was indirectly assessed by measuring the nitrite levels in the culture media using Griess reagent assay. Briefly, isolated cPBMCs were seeded at a density of 1×10^7^ cells/well in 96-well plates for 2 h. After incubation, the cells were treated with AAPH (10 mM) or LPS (100 ng/mL) for 24 h. The culture supernatant was harvested for nitrite assay. Each of 100 μL of culture media was mixed with an equal volume of Griess reagent (1% sulfanilamide, 0.1% naphthyl ethylenediamine dihydrochloride, and 5% phosphoric acid) and incubated at room temperature for 10 min, the absorbance was measured at 540 nm with a microplate reader.

### Measurement of prostaglandin E2 content in chicken peripheral blood mononuclear cells

Prostaglandin E2 (PGE_2_) level in cPBMCs was analyzed using kit purchased from Cayman Chemical Co., Ltd (Ann Arbor, MI, USA). Briefly, isolated cPBMCs were seeded at a density of 1×10^7^ cells/well in 96-well plates for 2 h. After incubation, cell culture supernatants were collected and assayed according to the manufacturer’s instruction. All samples were measured in triplicate. The total PGE_2_ in sample were expressed as pg per milliliter of culture supernatant.

### Statistical analysis

The data were analyzed by performing analysis of variances for completely randomized designs using the general linear model procedure of the SAS software program. Significant statistical differences among the various treatment group means were determined using Tukey’s honestly significant difference test. The effects of the experimental diets on response variables were considered to be significant at p<0.05.

## RESULTS

### Selected gene expression in chicken liver

**Figure 1:**
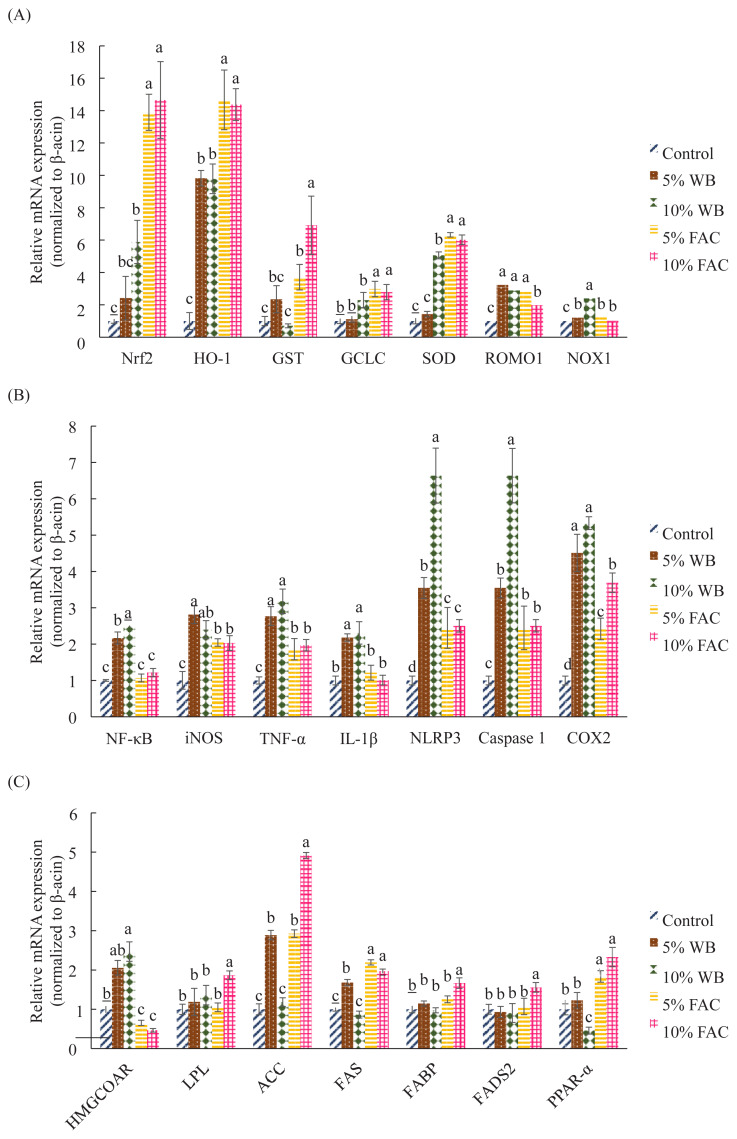
Effects of dietary replacement with wheat bran (WB) or fermented *Antrodia cinnamomea* (FAC) on the mRNA expression levels of selected genes in the chicken liver at 35 days. Values are expressed as the mean±standard deviation of eight replicates (n = 8). WB, wheat bran; FAC, fermented A. cinnamomea from Wang’s lab; Nrf2, nuclear factor erythroid-2-related factor; HO-1, heme oxygenase-1; GST, glutathione S-transferase; GCLC, glutamate cysteine ligase catalytic subunit C; SOD, superoxide dismutase; ROMO1, reactive oxygen species modulator 1; NOX1, NADPH oxidase 1; NF-κB, nuclear factor kappa-B; IL-6, interleukin-6; TNF-α, tumor necrosis factor-α; IL-1β, interleukin-1β; NLRP3, nod-like receptor protein 3; COX2, cyclooxygenase 2; HMGCoAR, 3-hydroxy-3-methyl-glutaryl-coenzyme A reductase; LPL, lipoprotein lipase; ACC, acetyl-CoA carboxylase; FAS, fatty acid synthase; FABP, fatty acid-binding proteins; PPARα, peroxisome proliferator activated receptor alpha; FADS2, fatty acid desaturase 2. ^a–d^ Means among groups without the same letter within the same sampling day are significantly different (p<0.05).

As shown in Figure 1A, distinct up-regulation of antioxidant genes were observed in 35-d-old chickens in 5% and 10% FAC group. Regarding 5% and 10% WB group, they shared similar pattern of antioxidant gene expression by and large, excluding that superoxide dismutase (*SOD*) in birds receiving 10% WB expressed higher than 5% WB group. On the contrary, reactive oxygen species modulator 1 (*ROMO1*) was up-regulated and showed comparable level in 5% WB, 10% WB, and 5% FAC as well, followed by 10% FAC and control group in the examining day. On the other hand, chickens in 10% WB group had maximal expression of NADPH oxygenase 1 (*NOX1*) among all groups; and other groups except for the corresponding control group presented similar expression level following 10% WB.

*NF-κB*, one of the main transcription protein regulating inflammatory signaling pathway, showed a roughly opposite pattern to *Nrf2* in 35 d-old chickens that 5% and 10% FAC group, accompanied by control group, demonstrated the lowest *NF-κB* expression than chickens receiving non-fermented WB in diet. Apart from control group, other groups expressed similar level of *NF-κB*, significant difference was only found between 10% WB and 5% FAC group (Figure 1B). The downstream molecules of *NF-κB* were shown in Figure 1B. For 35-d-old chicken, interleukin-6 (*IL-6*), tumor necrosis factor-α, and *IL-1β* demonstrated comparative trend with each other. Dietary replacement with WB had these genes been up-regulated, followed by FAC treatment group at either ratio, and then by control group. It’s noticeable that the direct and indirect activator of IL-1β, i.e. caspase-1 and nucleotide-binding domain, leucine-rich-containing family, pyrin domain-containing-3 (NLRP3, shared similar pattern to *IL-1β per se*, only that expression of *NLRP3* in 10% WB, 5% FAC, and 10% FAC groups increased significantly than the control group. Finally, 10% WB group also showed a relatively high, having no difference with 5% WB, and levels of *COX2* at 35 d, with 10% FAC, 5% FAC, and then the control group coming on next.

Changes of genes participating in lipid metabolism while receiving different diets were shown in Figure 1C. Except for 3-hydroxy-3-methyl-glutaryl-coenzyme A reductase (HMGCoAR) other six genes seem to express in similar pattern. In average, 10% FAC group showed significantly higher levels of these six genes. Although there was no difference among other four groups regarding lipoprotein lipase (LPL), fatty acid binding protein (FABP), and fatty acid desaturase 2 (FADS2), 10% WB inclusion would cause relatively severe reduction of acetyl-CoA carboxylase (ACC), fatty acid synthase (FAS), and PPARα. On the contrary, HMGCoAR increased significantly in birds consumed 10% WB diet, following by 5% WB, control, and then 5% and 10% FAC.

### Nuclear and cytoplasmic protein levels of Nrf2 and NF-κB

Since transcription factors activate downstream molecules in nucleus, and this occur only as they become functional protein. Accordingly, localization of Nrf2 and NF-κB were further determined by nuclear and cytosolic extracts using Western blot analysis to confirm the above mRNA results.

**Figure 2:**
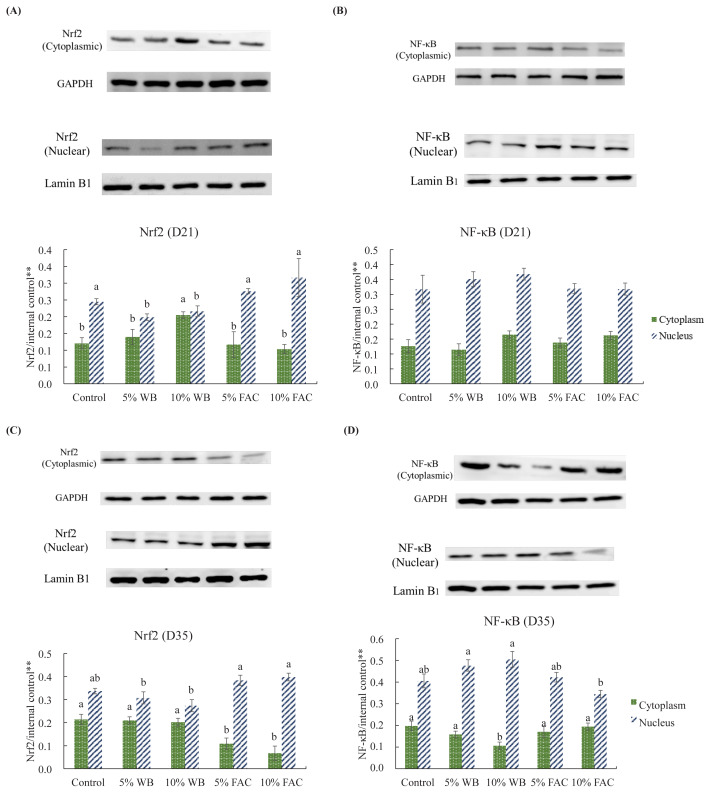
Effects of dietary replacement with wheat bran (WB) or fermented *Antrodia cinnamomea* from Wang’s lab (FAC) on Nrf2 and NF-κB p65 nuclear translocation in liver tissue of (A and C) 21d-old and (B and D) 35-d-old broiler chickens. Values are expressed as the mean±standard deviation of eight replicates (n = 8). ** The values under lanes indicate relative intensity of the band normalized to glyceraldehyde 3-phosphate dehydrogenase (for cytoplasmic protein normalization) or lamin B_1_ (for nuclear protein normalization) respectively. WB, wheat bran; FAC, fermented *A. cinnamomea* from Wang’s lab. ^a,d^ Means among groups without the same letter within the same sampling day are significantly different (p<0.05).

As shown in Figure 2A, nuclear Nrf2 expression in 5% and 10% WB markedly decreased comparing with the corresponding control group at d 21, which showed comparable level with 5% FAC and 10% FAC. Accompanied by the results of nuclear Nrf2, nuclear translocation of Nrf2 was inhibited in 10% WB that chickens in this group showed significant higher level of cytoplasmic Nrf2. At the same time (21 d), no difference was found NF-κB in either nucleus or cytoplasm among each group (Figure 2B).

For chickens aged 35-d, lower expression of cytoplasmic Nrf2 and higher level of nuclear Nrf2 in birds fed diet containing either replacement amount of FAC indicates a relative vigorous nuclear translocation of Nrf2 (Figure 2C). While three other groups showed commensurate Nrf2 protein in cytoplasm and nucleus. On the other hand, granted that none of the dietary replacement groups showed difference with their corresponding control group, it’s noteworthy that chickens receiving 10% FAC had lower NF-κB expressed in nucleus than other two WB included group, and cytoplasmic NF-κB expression was diminished significantly than other groups (Figure 2D).

### Cell viability upon immunological and oxidative stress

**Figure 3:**
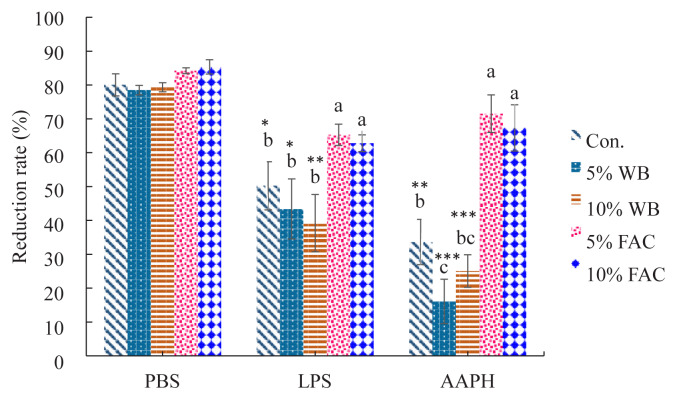
Effects of dietary replacement with product fermented *Antrodia cinnamomea* from Wang’s lab (FAC) on LPS and AAPH-induced cell death in peripheral blood mononuclear cells (PBMCs) isolated from chicken blood (35-d-old chicken). The percent dye reduction refers to chemical reduction of PrestoBlue, a resarzurin-based chemical reagent. The greater the magnitude of reduction, the greater the magnitude of metabolic activity and thus, presumably, the larger the number of viable cells. Values are expressed as the mean±standard deviation of eight replicates (n = 8). WB, wheat bran; FAC, fermented *Antrodia cinnamomea* from Wang’s lab; LPS, lipopolysaccharide; AAPH, 2,2′-Azobis(2-amidinopropane) dihydrochloride; PBS, phosphate-buffered saline. ^a–c^ Means among groups challenged by the same stimulus (PBS, LPS, or AAPH) without the same letter are significantly different (p<0.05). * p<0.05, ** p<0.01, and *** p<0.001 were considered significant for LPS- or AAPH-treated sample versus its corresponding PBS-treated sample.

Cell tolerance against negative impacts, i.e. oxidative and immunological challenges in this study, was examined by incubating cPBMCs isolated from chickens in each treatment groups with AAPH and LPS. As shown in Figure 3, except for 5% and 10% FAC group, cell viability in other groups were all significantly compromised under either LPS or AAPH challenge; among them, as comparing with the corresponding PBS-treated group, 10% WB group showed the lowest tolerance upon both challenges (p<0.005 or p<0.0001). Furthermore, 5% and 10% FAC exhibited significantly higher viability in comparison with other groups exposing to the same stimulus, suggesting a potential protective effect exerted by FAC.

### H_2_O_2_ and NO levels in chicken peripheral blood mononuclear cells challenged by LPS or AAPH

**Figure 4:**
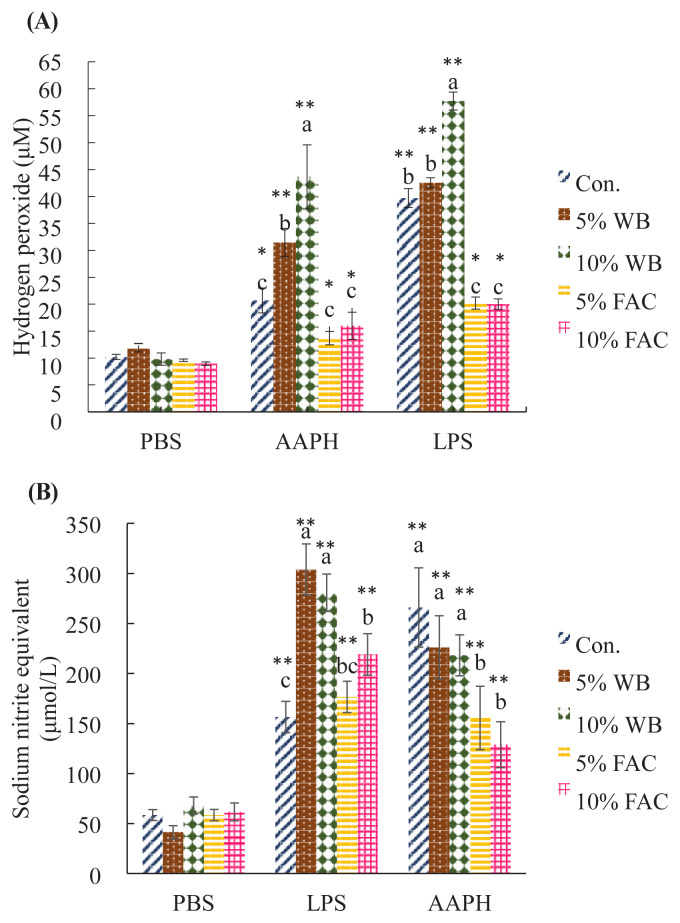
Effects of dietary replacement with wheat bran (WB) or fermented *Antrodia cinnamomea* from Wang’s lab (FAC) on (A) hydrogen peroxide and (B) nitric oxide (NO) production in LPS- and AAPH-induced chicken peripheral blood mononuclear cells (cPBMCs) in 35 d-old chicken. Values are expressed as the mean±standard deviation of eight replicates (n = 8). WB, wheat bran; FAC, fermented *Antrodia cinnamomea* from Wang’s lab; LPS, lipopolysaccharide; AAPH, 2,2'-Azobis(2-amidinopropane) dihydrochloride; PBS, phosphate-buffered saline. ^a–c^ Means among groups challenged by the same stimulus (PBS, LPS, or AAPH) without the same superscript letter are significantly different (p<0.05). * p<0.05 and ** p<0.01 were considered significant for LPS- or AAPH-treated sample versus its corresponding PBS-treated sample.

Since external challenges often lead to oxidative stress in cells, study further proceeded to investigate LPS- and AAPH-induced intracellular hydrogen peroxide (H_2_O_2_) and NO content in cPBMCs. Figure 4A shows the effects of dietary replacement with WB and FAC on AAPH- and LPS-induced hydrogen peroxide level in cPBMCs. H_2_O_2_ level in cells of all groups increased significantly upon either AAPH or LPS challenge, however, cPBMCs in chickens of different group showed divergent capacity of homeostatic regulation against both challenges. Concerning cells confronting the same stimulus among groups, H_2_O_2_ content in cPBMCs of chickens fed 10% WB replacement diet rose significantly higher than other groups, followed by 5% WB group. Furthermore, chickens in 5% and 10% FAC had similar production of H_2_O_2_ level under stimuli, and both of them showed significantly lower H_2_O_2_ level than WB treated groups yet numerically lower than the level in control group upon AAPH challenge.

Similarly, LPS and AAPH both caused NO level signifi cantly increased in 5% and 10% WB groups, and cells in control group showed comparable NO content to both two WB groups upon AAPH challenge, even though it presented the lowest NO amount under LPS inducement. Moreover, 5% and 10% FAC had the lowest NO production after incubating with AAPH, and with LPS incubation, there was no difference in NO production between these two groups (Figure 4B).

### Prostaglandin E2 level in chicken peripheral blood mononuclear cells

**Figure 5:**
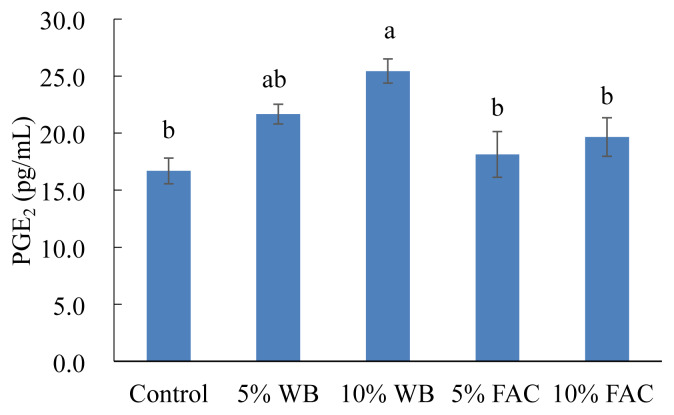
Effects of dietary replacement with wheat bran (WB) or fermented *Antrodia cinnamomea* from Wang’s lab (FAC) on the prostaglandin E2 (PGE_2_) production in the chicken peripheral blood mononuclear cells (cPBMCs) at 35 d. Values are expressed as the mean±standard deviation of eight replicates (n = 8). WB, wheat bran; FAC, fermented *Antrodia cinnamomea* from Wang’s lab. ^a,b^ Means without the same letter are significantly different (p<0.05).

Figure 5 shows the effects of dietary replacement with WB or FAC on PGE_2_ level in cPBMCs. No significant difference was found between the control group with 5% WB, 5% FAC, or 10% FAC group. Nevertheless, 10% WB inclusion in diet remarkably increased PGE_2_ level in comparison with other groups except for 5% WB group.

## DISCUSSION

Since the interaction of an organism with its nutrition sources is an intimate and complicated physiological event that is typically relying on multiple systems, even involves some minor microflora working in concert, targeting deeper and more original mechanisms become necessary. Thanks to the development of the field of molecular biology, researchers have been able to study the impact of diet on the animals at the clearer molecular level. By dissecting the mechanism of the effects of nutrients or the effects of a nutritional regime, this approach provide deeper insight into a more thorough process while changing diet for animals, it can also be used to evaluate the physiological effects of specific nutrients. The regulatory control mechanisms of these processes can be based on all levels from genetics and gene expression to the feedback of specific metabolites.

Recent research on AC has made great leap forward in re vealing its active components and their underlying molecular mechanism in cell lines or mammal model [14] found that fermented culture broth of AC significantly inhibited LPS-induced NO and PGE_2_ production and attenuated their corresponding genes, inducible nitric oxide synthase (*iNOS*) and *COX2*, by impeding NF-κB activation; at the same time, reduced production of cytokine in LPS-challenged group further supported the restricting NF-κB activity. Correspondingly, our results showed that chickens receiving diet containing non-fermented WB had relatively higher expression of NF-κB, iNOS, and COX2. High amount of non-starch polysaccharides (NSP) in WB are undesirable since NSP may induce gut inflammation in broiler chickens [18], which may associate with NF-κB activation [19]. Moreover, it’s noteworthy that *NLRP3* and *Caspase 1* genes expressed similar pattern in the same group. NLRP inflammasome is a multiprotein complex. NLRP3 is of importance lies on the fact that animals are not always exposed to pathogens; however, under the stimulants such as stress, damage associated molecular patterns would be released and activate inflammasome that induce maturation of pro-inflammatory cytokines. The subsequent cytokines, with those stimulated by NF-κB, would continuously activate NF-κB pathway, thus forming a positive auto-regulatory loop that can amplify the inflammatory response [20]. Therefore, the increased level of NLRP3 and caspase 1 in two WB inclusion groups suggested that dietary supplementation of 5% to 10% WB may trigger stress response in broiler chickens, and thus activate NF-κB inflammatory pathway.

On the other hand, induction of Nrf2-regulated phase II antioxidant genes is considered as the core cellular defense in response to oxidative stress [10]. These genes include GST and GCLC, both of which are indispensable molecules maintaining the homeostasis of GSH antioxidant system. Moreover, it’s noticeable that HO-1 was increased with significant level in the FAC groups as well. Previous literatures in mammal studies have proved the important role of HO-1 activation via Nrf2 pathway in countervailing inflammation [21]. ROMO1 and NOX1 are regarded as positive regulators in intracellular ROS production [22]. It has been proven that increased *ROMO1* expression, in response to external stress, enhances cellular ROS levels; its expression is also essential for cancer cell proliferation [22]. NOX1 is one of the vital components of the enzymes catalyzing the production of O_2_^−^ and H_2_O_2_, both of which are members of ROS. Therefore, the amelioration of these two gene expressions in the current study were in line with the activation of Nrf2 and its downstream effects.

Currently, the antagonized role of Nrf2 pathway in NF-κB- modulated inflammation is gradually applied to animal science. For example, decreased SOD, catalase (CAT) and GSH-Px activities along with suppressed Nrf2 and augmented NF-κB expression in quails exposed to heat stress were improved by dietary supplementation with resveratrol and curcumin [23], two phytochemicals well proved to have cytoprotective effects in medical research. In order to confirm the results of mRNA expression, Nrf2 and NF-κB protein level in the nucleus and cytoplasm of liver cell were analyzed. In unstimulated form, both transcription factors are generally sequestered by their suppressor in cytoplasm and be kept in low levels in nucleus. While activation, they would not be degraded by their oppressors, and they will thus translocate to the nucleus and to activate their downstream gene expression. According to our results, increased Nrf2 protein in the nucleus and the corresponding decrease in the cytoplasm suggested that in comparison with other three groups, 5% and 10% FAC supplementation drove the activation of Nrf2 protein. By contrast, NF-κB activation were inhibited especially in 10% FAC group in terms of relatively low level in the nucleus; and for chickens obtained diet containing non-fermented WB, relatively higher expression of cytoplasmic NF-κB and its downstream genes such as IL-6 and IL-1β suggested that chickens may be in a more stressful status, or even suffer from a certain level of inflammation.

In our study, the precursor and activator of *IL-1β*, *NLRP3*, and *caspase 1*, were up-regulated especially in 10% WB group, implying more stressed condition of chickens. Previous literature has shown that activation of *IL-1β* and *caspase1* is ROS-dependent [24]. In a positive feedback loop, IL-1β promotes intracellular accumulation of ROS by uncoupling antioxidant enzyme, especially SOD. This stance partially supports the current study, in which relative higher *NLRP3* accompanied by increased *caspase 1* and *IL-1β* in 10% WB may lead to suppressed *SOD*; whilst, cells which were isolated from chickens received 10% WB diet were susceptible to extracellular challenge in terms of higher H_2_O_2_ and NO production may also buttress the above standpoint. NO is a product from three types of NOS; however, apart from two of them constitutively expressed, iNOS only expressed upon cell activation [25]. Our study showed that as being stimulated by AAPH and LPS, which are responsible for inducing free radical and activating inflammation, H_2_O_2_ was increased especially in 10% WB group, followed by 5% WB; and despite not completely consistent NO level, chickens received FAC in diet showed relatively low NO production after extracellular stimulation. It was reported that the fate of cell depends on the balance between activities of classes of gene, including those encoding for the expression of inflammatory related molecules (such as interleukin, NO, and PGE_2_) [26], higher cell viability observed in FAC inclusion groups may thus further support the results of gene and protein expression level.

In another manuscript, fatty acid profile in meat indicated that FAC supplementation may increase the ratio of monounsaturated and saturated fatty acids. This may result from the up-regulated genes for enzymes involved in FA synthesis (Figure 1C), in which FAS and FADS2 were expressed in equal high level. FAS plays a central role in *de novo* lipogenesis in animal body, and FADS2 is responsible for be a rate limiting step in *de novo* long-chain polyunsaturated fatty acids synthesis [27]; these support the results found in fatty acid composition in chicken muscle. Moreover, it was reported that different diet can alter the expression of PPARs in broiler livers [28], and as the expression of PPARα increased, fatty acid β-oxidation would be enhanced [29]. These could attribute to the repressed triglyceride (TG) level in chickens receiving FAC in diet. Moreover, since cholesterol synthesis is a highly regulated pathway subjecting to transcriptional modulation, the decreased TG level that is indirectly correlated to the cholesterol and high density lipoprotein metabolism was thus hypothized to be attributed to not only PPARα, but also the suppressed HMGCoAR regulating cholesterol synthesis and the indirect stimulation of LPL expression [6]. Several studies found that polyphenols were able to suppressed cholesterol synthesis through inhibition of HMGCoAR expression [30]. It’s noteworthy that the lipid-regulation mechanism of types of polyphenol in AC hasn’t yet to be studied, suggesting a new sphere worthy to be developed.

It was reported that activated PPARα is capable of repress ing inflammatory genes, such as caspase, COX2 and IL-6, by promoting the cytoplasmic inhibitor of NF-κB, thus suppressing NF-κB entering nucleus to activate downstream molecules [31]. For those NF-κB related inflammatory genes, they indeed showed approximately opposite pattern with PPARα, FAS, and ACC. However, it’s interesting to find that in our study, PPARα, FAS, and ACC were expressed in similar pattern with antioxidant genes regulated by Nrf2. These seem to be associated with the indirectly antioxidant action of PPARα by upregulation of SOD, CAT, HO-1, and even GSH-related genes and inhibit NADPH oxidase (*NOX*) gene activation and consequently ROS generation [24]. Furthermore, results in present study seem to in line with previous study demonstrating that augmentation of PPARα could increase HO-1 and decrease caspase expression [31]. Since PPARα, similar to Nrf2 and NF-κB, is highly sensitive to redox perturbations [6], and that it’s important to solve oxidative stress and to improve lipid profile in chicken meat [32].

**Figure 6:**
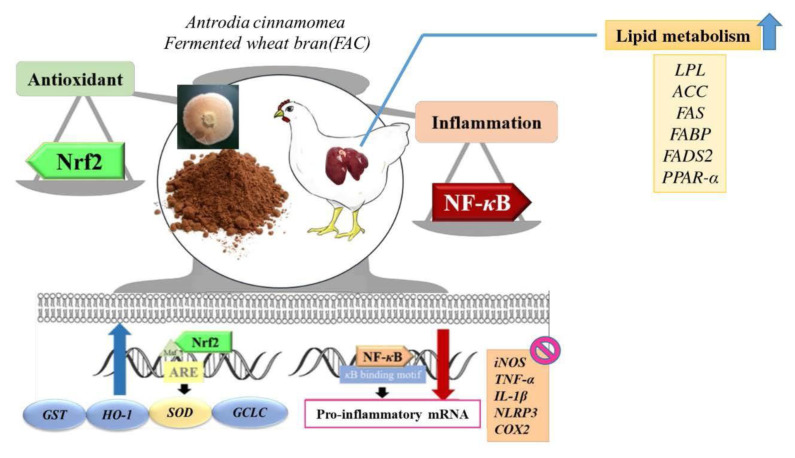
Potential crosstalk among antioxidant, inflammatory, and lipid metabolism pathways in fermented *Antrodia cinnamomea* supplemented broiler.

In conclusion, our study addresses possible interplay among oxidative, inflammatory, and lipid metabolism pathways in broiler chickens through WB and FAC. Dietary supplementation of wheat bran promoted the activation of molecules involved in NF-κB pathways in chickens at gene level, which further supported by relatively high NF-κB protein ratio in nucleus. Meanwhile, chickens with FAC rather showed opposite results in terms of relatively higher expression of Nrf2 antioxidant pathways. The improved defense mechanisms summarized above are in line with suppressed H_2_O_2_ and NO production, and even cell viability challenged by extracellular reagents. Moreover, increased expression of lipid-related genes like PPARα, FAS, and FADS2; and inhibited HMGCoAR buttress better FA saturated-ratio and serum lipid parameters in FAC groups in our separated manuscript. These lipid-regulation are promising links with Nrf2 and NF-κB regulatory pathways in terms of similar patterns of expression in Nrf2 regulated genes and approximately opposite pattern with NF-κB related inflammatory genes. The above findings demonstrate potential molecular targets to dissect ROS-related complications in broiler chickens, and to develop strategies to manipulate the balance among Nrf2, NF-κB, and PPARα responses under specific conditions in modern rearing environment (Figure 6).

## Figures and Tables

**Table 1:** Ingredients and chemical composition of the experimental diets for broilers

Items	Starter diet (1 to 21 d)					Finisher diet (22 to 35 d)				
	
	Control	5% WB	10% WB	5% FAC	10% FAC	Control	5% WB	10% WB	5% FAC	10% FAC
	----------------------------------------------- g/kg -----------------------------------------------									
Corn, yellow	474.4	406.9	339.5	413.1	351.8	520.6	453.2	385.7	459.3	398.1
Soybean meal (CP 44%)	178.5	166.0	153.6	160.7	143.0	103.8	91.3	78.9	86.1	68.3
WB	0	50	100	0	0	0	50	100	0	0
FAC	0	0	0	50	100	0	0	0	50	100
Soybean oil	1.0	29.6	58.3	28.5	56.0	5.7	34.3	62.9	33.2	60.7
Full fat soybean meal	300	300	300	300	300	330	330	330	330	330
Calcium carbonate	16.0	16.0	16.1	16.1	16.1	13.3	13.3	13.3	13.3	13.3
Monocalcium phosphate	18.0	18.5	18.8	18.5	18.8	16.2	16.5	16.7	16.5	16.8
L-lysine-HCl	1.6	2.2	2.8	2.4	3.2	0.9	1.5	2.1	1.6	2.4
DL-methionine	3.7	4.1	4.5	4.2	4.6	3.1	3.5	3.9	3.5	3.9
NaCl	4.0	3.7	3.8	3.7	3.8	3.7	3.7	3.7	3.7	3.7
Choline-Cl	0.8	0.8	0.8	0.8	0.8	0.8	0.8	0.8	0.8	0.8
Vitamin premix1)	1	1	1	1	1	1	1	1	1	1
Mineral premix2)	1	1	1	1	1	1	1	1	1	1
Total	1,000	1,000	1,000	1,000	1,000	1,000	1,000	1,000	1,000	1,000
Calculated nutrient value										
ME (kcal/kg)	3,050	3,050	3,050	3,050	3,050	3,175	3,175	3,175	3,175	3,175
Crude protein (g/kg)	230	230	230	230	230	210	210	210	210	210
Calcium (g/kg)	10.5	10.5	10.5	10.5	10.5	9.0	9.0	9.0	9.0	9.0
Total phosphorus (g/kg)	7.7	7.5	7.4	7.5	7.5	7.1	7.0	7.0	7.0	7.1
Available phosphorus (g/kg)	5.0	5.0	5.0	5.0	5.0	4.5	4.5	4.5	4.5	4.5
Lysine (g/kg)	14.3	14.3	14.3	14.3	14.3	12.5	12.5	12.5	12.5	12.5
Methionine+cystein (g/kg)	10.7	10.7	10.7	10.7	10.7	9.6	9.6	9.6	9.6	9.6
Analyzed nutrition value										
Crude protein (g/kg)	231	234	232	231	232	213	211	212	212	212
Crude fat (g/kg)	78.6	76.4	76.8	76.6	76.8	85.1	84.6	84.1	84.8	85.3

CP, crude protein; WB, wheat bran; FAC, products solid-state fermented wheat bran by *Antrodia cinnamomea* for 16 days; ME, metabolizable energy.

1) Supplied per kg of diet: Vit A 15,000 IU; Vit. D_3_ 3,000 IU; Vit. E 30 mg; Vit. K_3_ 4 mg; riboflavin 8 mg; pyridoxine 5 mg; Vit. B_12_ 25 μg; Ca-pantothenate 19 mg; niacin 50 mg; folic acid 1.5 mg; biotin 60 μg.

2) Supplied per kg of diet: Co (CoCO_3_) 0.255 mg; Cu (CuSO_4_·5H_2_O) 10.8 mg; Fe (FeSO_4_·H_2_O) 90 mg; Zn (ZnO) 68.4 mg; Mn (MnSO_4_·H_2_O) 90 mg; Se (Na_2_SeO_3_) 0.18 mg.

**Table 2:** Primers used for quantitative polymerase chain reaction analysis

Gene	Forward primer (from 5’ to 3’) everse primer (from 5’ to 3’)
*β-actin*	CTGGCACCTAGCACAATGAA ACATCTGCTGGAAGGTGGAC
*HO-1*	AGCTTCGCACAAGGAGTGTT GGAGAGGTGGTCAGCATGTC
*NOX1*	CAATGCAGCACTCCACTTTG GACAAGATCTCCGCAAGACC
*GST*	AGTCGAAGCCTGATGCACTT TCTAGGCGTGGTTTCCTTTG
*GCLC*	CAGCACCCAGACTACAAGCA CTACCCCCAACAGTTCTGGA
*SOD*	GCCACCTACGTGAACAACCT AGTCACGTTTGATGGCTTCC
*Nrf2*	GGAAGAAGGTGCTTTTCGGAGC GGGCAAGGCAGATCTCTTCCAA
*ROMO1*	AGCCCAGCTGCTTCGACAGAGT CGTCCTCTCATGCCGATCCTGA
*NF-κB*	GAAGGAATCGTACCGGGAACA CTCAGAGGGCCTTGTGACAGTAA
*IL-1β*	GCTCTACATGTCGTGTGTGATGAG TGTCGATGTCCCGCATGA
*IL-6*	AGGACGAGATGTGCAAGAAGTTC TTGGGCAGGTTGAGGTTGTT
*COX2*	TGTCCTTTCACTGCTTTCCAT TTCCATTGCTGTGTTTGAGGT
*NLRP3*	GGTTTACCAGGGGAAATGAGG TTGTGCTTCCAGATGCCGT
*Caspase1*	GATACGTGACTCCATCGACCC CTTCTTCAGCATTGTAGTCC
*HMGCoAR*	TGTTGTAAGGCTGCCCTCTG TAGGCGGGCAAACCTACTTG
*LPL*	AGTCAGAGTGAAGTCAGGCGAAAC CTGCTCCAGGCACTTCACAAATA
*ACC*	CTGATGGTCTTTGCCAACTGGA CACGATGTAGGCACCAAACTTGA
*FAS*	TCAGGGTGTTCTGGAATGCAA
	AATCCTGGTGGGCAATCGTAG
*FABP*	ATGAGCTTCACTGGAAAGTACGAG TCTTGATGTCCTTACCCTTCTGG
*PPARα*	TGCACTGGAACTGGATGATAGTGA TCCTACATTTACAAGACCAGGACGA
*FADS2*	TCACTTGTGGAGGTAAGCATC GGCGAGAAAGGAGAGGAGTC

*β-Actin* (NCBI GenBank no: X00182.1); *HO-1*, heme oxygenase -1 (NCBI GenBank no: X56201.1); *NOX1*, NADPH oxygenase 1 (NCBI GenBank no: NM_001101830.1); *GST*, glutathione S-transferase (NCBI GenBank no: L15386.1); *GCLC*, glutamate-cysteine ligase, catalytic subunit (NCBI GenBank no: XM_419910.3); *SOD*, superoxide dismutase (NCBI GenBank no: NM_204211.1); *Nrf2*, nuclear factor (erythroid-derived 2)-like 2 (NCBI GenBank no: NM_205117.1); *ROMO1*, reactive oxygen species modulator protein 1 (NCBI GenBank no: NM_001198821.1); *NF-κB*, nuclear factor of kappa light polypeptide gene enhancer in B-cells p65 (NCBI GenBank no: NM_205134); *IL-1β*, interleukin 1-beta (NCBI GenBank no: NM_204524); *IL-6*, interleukin 6 (NCBI GenBank no: NM_204628); *COX2*, cyclooxygenase 2 (NCBI GenBank no: NM_001167718.1); *NLRP3*, nod-like receptor protein 3 (NCBI GenBank no: NC_006092.4); *Caspase1* (NCBI GenBank no: NC_006106.4); *HMGCoAR*, 3-hydroxy-3-methyl-glutaryl-coenzyme A reductase (NCBI GenBank no: NM_204485); *LPL*, lipoprotein lipase (NCBI GenBank no: NC_006127.4); *ACC*, acetyl-CoA carboxylase (NCBI GenBank no: NC_006106.4); *FAS*, fatty acid synthase (NCBI GenBank no: NC_006105.4); *FABP*, fatty acid-binding proteins (NCBI GenBank no: NC_006091.4); *PPARα*, peroxisome proliferator activated receptor alpha (NCBI GenBank no: NC_006088.4); *FADS2*, fatty acid desaturase (NCBI GenBank no: NC_006092.4).
